# Association of Three Genetic Loci with Molar Incisor Hypomineralization in Polish Children

**DOI:** 10.3390/jcm13030857

**Published:** 2024-02-01

**Authors:** Marta Milona, Andrzej Ciechanowicz, Karolina Węsierska, Magda Gońda-Domin, Alicja Zawiślak, Anna Jarząbek, Marta Sobalska-Kwapis, Justyna Jarczak, Renata Gruszka, Dominik Strapagiel, Joanna Janiszewska-Olszowska, Katarzyna Grocholewicz

**Affiliations:** 1 Department of Hygiene and Epidemiology, Pomeranian Medical University, 70-111 Szczecin, Poland; marta.milona@pum.edu.pl; 2 Department of Clinical and Molecular Biochemistry, Pomeranian Medical University, 70-111 Szczecin, Poland; 3 Independent Researcher, 70-236 Szczecin, Poland; kwesierska@yahoo.com; 4 Independent Researcher, 70-356 Szczecin, Poland; gondzik@wp.pl; 5 Department of Interdisciplinary Dentistry, Pomeranian Medical University, 70-111 Szczecin, Poland; alicja.zawislak@pum.edu.pl (A.Z.); joanna.janiszewska.olszowska@pum.edu.pl (J.J.-O.); katarzyna.grocholewicz@pum.edu.pl (K.G.); 6 Laboratory of Paediatric Dentistry, Pomeranian Medical University, 70-111 Szczecin, Poland; anna.jarzabek@pum.edu.pl; 7 Biobank Lab, Department of Oncobiology and Epigenetics, Faculty of Biology and Environmental Protection, University of Lodz, 90-237 Lodz, Poland; marta.sobalska@biol.uni.lodz.pl (M.S.-K.); dominik.strapagiel@biol.uni.lodz.pl (D.S.); 8 Regenerative Medicine Laboratory, Medical University of Warsaw, 02-097 Warszawa, Poland; justyna.jarczak@wum.edu.pl; 9 Department of Molecular Biotechnology and Genetics, Faculty of Biology and Environmental Protection, University of Lodz, 90-237 Lodz, Poland; renata.gruszka@biol.uni.lodz.pl

**Keywords:** genetic polymorphism, molar incisor hypomineralization, replication study

## Abstract

Background: Molar incisor hypomineralization (MIH) is a qualitative, demarcated enamel defect of hypomineralization affecting one to four first permanent molars, often with incisor involvement. Its etiology is complex. However, evidence suggests the influence of genetic factors, potentially including the single nucleotide polymorphisms (SNPs) rs2889956, rs4811117 and rs13058467, which were previously linked to MIH in a genome-wide association study of German children. The aim was to replicate analyses of possible associations between the SNPs and molar incisor hypomineralization in Polish children. Methods: The final study group consisted of 778 children aged 126–168 months old. Saliva samples were taken, and genomic DNA was extracted and genotyped using beadchip microarrays. Results: Among the 778 subjects, there were 68 (8.7%) subjects with MIH and 710 (91.3%) subjects without MIH. There were no significant differences in distributions in age, sex, or the frequency of caries in permanent dentition between the MIH and non-MIH groups. The rs2889956, rs4811117, and rs13058467 genotype distributions in the studied group conformed to the expected Hardy–Weinberg equilibria, and there were no significant differences in the distributions of their alleles or genotypes between the MIH and non-MIH groups. Conclusion: Our replication study did not confirm highly significant associations between the single nucleotide polymorphisms rs2889956, rs4811117, and rs13058467 with molar incisor hypomineralization in Polish children.

## 1. Introduction

Molar incisor hypomineralization (MIH) has been defined as a qualitative, demarcated enamel defect of hypomineralization affecting one to four first permanent molars (FPM) [1]. However, permanent incisors (PI) can also often be affected; hence, the name MIH. Other permanent teeth can be affected, and in 2021, Keverekidou et al. proposed a description of the presence of lesions characteristic of MIH in permanent teeth other than FPM and PI as “hypomineralization of other permanent teeth” (HOPT). They reported that patients with MIH can have an up to three times higher probability of HOPT than patients without MIH [2]. The European Academy of Paediatric Dentistry (EAPD), in its most recent update of 2022, has included enamel defects occurring on second primary molars, premolars, second permanent molars, or canine cusps as possible concomitant diagnostic criteria of MIH [1].

According to the EAPD, the definitive diagnostic criterion of MIH consists of at least one or more first permanent molars with one of the following characteristics: demarcated enamel opacity (of at least 1 mm in diameter), posteruptive enamel breakdown, atypical restoration, or atypical extraction (due to related phenomena) [1,2]. 

MIH is classified as mild when there are demarcated opacities without posteruptive enamel breakdown or as severe when posteruptive enamel breakdown has occurred [3]. Clinically, MIH is present on affected teeth as clearly demarcated opacities of various colors (white, creamy or yellow to brownish) [1]. (Only defects larger than 1 mm should be considered). Some research has shown that teeth affected by MIH may be more sensitive to thermal stimuli or give a shooting pain during brushing. Subsequently, this can therefore facilitate plaque accumulation and the development of dental caries [4]. 

A meta-analysis based on 116 observational studies indicated a world-wide pooled prevalence of MIH of 13.5%, with no significant differences found between geological continents. For comparison, Lopes et al. found the highest MIH prevalence in America at 15.3% and the lowest in Asia (10.7%), while the MIH frequency in Europe was 14.4% [5].

MIH etiology is complex, multifactorial, and still not fully elucidated, although evidence suggests the influence of both environmental and genetic factors [6,7]. Results of a recent meta-analysis performed by Juarez-Lopez et al. clearly revealed associations between MIH and phenotypic factors, such as a history of illness during pregnancy, low birth mass, general illness in childhood, antibiotic use, and high fever during early childhood [8]. On the other hand, familial inheritance studies [9,10] or candidate-gene identification of MIH-associated single nucleotide polymorphisms (SNPs) (as recently reviewed by da Silva Figueira et al. [7] and by Teixeira et al. [11]) strongly suggest a genetic contribution.

As far as we know, the article by Kühnisch et al. provides the only genome-wide association study (GWAS) currently available that analyzed the relationship between genetic polymorphisms and susceptibility to molar incisor hypomineralization [12]. The study group in this GWAS consisted of 668 children (mean age: 10.2 years old) born in Munich, Germany, and a total of 2,013,491 SNPs from each child were available for analysis. The GWAS results revealed several SNPs on chromosomes 4, 9, 16, 20, and 22 with low *p*-values suggestive of an association with MIH, but none of these reached the genome-wide assigned significance threshold of <5 × 10^−8^ [13]. However, using a threshold *p*-value of <1 × 10^−6^, the authors suggested that polymorphisms at some of these chromosomal loci might possibly be associated with MIH risk, among them rs13058467, located near the protein-coding *SCUBE1* gene at chromosome 22q11.22 with *p* < 3.72 × 10^−7^ [12]. Therefore, we decided to replicate analyses of these possible associations between the SNPs at these chromosomal loci and molar incisor hypomineralization in children of Polish descent.

## 2. Materials and Methods

The study group consisted of children of Polish descent recruited between 2019 and 2022 for “Task 05”, part of the Program “Regional Initiative of Excellence”, from the Polish Minister of Science and Higher Education (Project no. 002/RID/2018/19). “Task 05” aimed to carry out molecular analyses of oral microbiomes and DNA microarray-based genome polymorphisms from saliva samples and to evaluate their distributions with respect to predisposition to caries of permanent dentition in children. 

The children were recruited in primary schools that were part of the system of obligatory general education in Szczecin (the largest city in the Western Pomeranian region of Poland). The study was performed according to the latest Declaration of Helsinki. Approval was obtained from the Bioethics Committee of the Pomeranian Medical University in Szczecin. Parents were informed about study objectives and gave signed informed consent before their children’s’ participation. 

For each subject, a clinical dental examination was performed in order to collect detailed information regarding oral health status, including the assessment of caries and molar incisor hypomineralization. The dental examination was performed by four trained, qualified, and experienced dentists who were specialists in pediatric dentistry or orthodontics. The children were examined in a sitting position in artificial light, using a dental mirror and a probe. The deciduous and permanent teeth present in the oral cavity were marked on a standard chart; the numbers of deciduous and permanent teeth were computed. Caries was diagnosed if a dental cavity could be stated based on visual–tactile examination [14]. All dental cavities, fillings, secondary caries, fissure sealers, and missing teeth were marked on the chart. The Decayed Missing Filled Teeth (DMFT) Index was calculated for every child as the sum of decayed (D) missing due to caries (M) and filled teeth (F). Additional incidences of caries were defined, according to World Health Organization (WHO) caries diagnostic criteria, with a value of DMFT ≥ 1 [15]. Molar incisor hypomineralization was diagnosed according to the European Academy of Pediatric Dentistry [1] if at least one molar was present with a demarcated enamel opacity or hypomineralization (diameter greater than 1 mm). Teeth affected were noted, and the appearance of lesion(s) was described. 

Before the dental examination, a sample of saliva was collected from each subject, placed in a specialized DNA collector (GeneFiX™ 2 mL DNA Collector; GFX-02; Isohelix, Cell Projects, Maidstone, UK), and stored at room temperature until the extraction process. Genomic DNA was extracted from 900 µL of saliva (using a GeneFix Saliva Prep DNA Isolation Kit, Isohelix). The precipitated DNA was rehydrated with 100 µL of nuclease-free water. A total of 792 DNA samples from patients were quantified (using Quant-iT™ dsDNA Broad Range Assay Kits; Invitrogen™, Carlsbad, CA, USA). The DNA sample concentrations were normalized to 50 ng/µL [16]. DNA samples were genotyped for 567,218 SNPs using beadchip microarrays (Infinium CoreExome-24 v1-4; Illumina, San Diego, CA, USA) according to the protocol provided by the manufacturer.

From 800 children initially recruited for “Task 05”, part of the Program “Regional Initiative of Excellence” (Polish Minister of Science and Higher Education, Project no. 002/RID/2018/19), 22 children were excluded due to poor quality of the DNA samples extracted or missing genotypes of SNPs previously suggested to be associated with MIH [12]. Our final study group therefore consisted of 778 children aged 126 to 168 months (362 females; 416 males). This final study group was, following assessment of MIH, divided into those with MIH (the MIH group) and those without MIH (the non-MIH group) for further, including genetic, analyses.

Quantitative data are presented as medians (with ranges) and were analyzed using Mann–Whitney U tests. Possible divergence of genotype frequencies from Hardy–Weinberg equilibria and the differences between groups in terms of distributions of sex, caries frequency, and frequencies of genotypes and alleles were assessed using χ2 tests (with Yates corrections if necessary). The frequency distribution of genotypes in dominant, recessive, or additive modes of inheritance for minor allele were compared between groups using logistic regression. Statistical significance was defined as *p* < 0.05 with or without Bonferroni correction. All data were analyzed using a commercial data analysis software system (Dell Statistica, version 13. Dell Inc. 2016, software.dell.com, accessed on 20 July 2023).

## 3. Results

Single nucleotide polymorphisms from three of the five chromosomal loci suggested in the article by Kühnisch et al. [12] as possibly associated with MIH (with *p*-values < 1 × 10^−6^) were found among the 567,218 SNPs on the microarrays used for genotyping. 

In the study group, for rs2889956, there were 380 (48.8%) GG homozygotes, 324 (41.6%) GA heterozygotes, and 74 (9.5%) AA homozygotes, with a minor A allele frequency of 30.3%. For rs4811117, there were 652 (83.8%) GG homozygotes, 124 (15.9%) GT heterozygotes, and 2 (0.3%) TT homozygotes, with a minor T allele frequency of 8.2%. For rs13058467, there were 653 (83.9%) TT homozygotes, 122 (15.7%) TC heterozygotes, and 3 (0.4%) CC homozygotes, with a minor C allele frequency of 8.2%.

The rs2889956, rs4811117, and rs13058467 genotype distributions in the final study group conformed to the expected Hardy–Weinberg equilibria (*p* = 0.683, *p* = 0.122 and *p* = 0.283, respectively). Among the 778 subjects, there were 68 (8.7%) subjects with MIH (the MIH group) and 710 (91.3%) subjects without MIH (the non-MIH group). There were no significant differences in the distributions of age, sex, or the frequency of caries in permanent dentition between the MIH and non-MIH groups (Table 1). 

No significant differences in the distributions of rs2889956, rs4811117, and rs13058467 alleles were found between the MIH and non-MIH groups. No significant differences in the frequency distributions of rs2889956 and rs13058467 genotypes were found between the MIH and non-MIH groups. However, there was a significant difference in the frequency distribution of rs4811117 between these two groups. No significant associations between rs2889956, rs4811117, or rs13058467 and MIH were shown in dominant, recessive, or additive modes of inheritance of the minor alleles (Table 2).

## 4. Discussion

To the best of our knowledge, the genome-wide association study by Kühnisch et al. is the only analysis of this type, so far, devoted to the identification of chromosomal loci associated with susceptibility to molar incisor hypomineralization [12]. Although the authors did not discover any MIH locus with an association *p*-value below the genome-wide significance *p*-value threshold of 5 × 10^−8^, commonly accepted for a standard GWAS [13], they did identify five chromosomal loci as possibly linked to MIH using a threshold *p*-value of 1 × 10^−6^ [12]. These MIH-suggestive loci were found on chromosome 4 tagged by rs17650401; chromosome 9 with rs2889956, rs13288553, and rs7028167 as the top tagging SNPs; chromosome 16 (in a region flanked by rs1969527 and rs1126179); chromosome 20 between the locations of rs4811117 and rs1123093; and chromosome 22 tagged by five SNPs, including rs13058467, rs11703432, rs11704836, rs11704919, and rs2071725 [12]. 

It is worth noting that, in the above chromosomal regions identified by Kühnisch et al., there were no loci found for genes with polymorphisms so far associated with MIH risk as gene candidates, including genes encoding proteins involved in amelogenesis, immune response, or xenobiotic detoxification [7,11].

Our analyses have replicated some association studies from the GWAS by Kühnisch et al. performed in an independent cohort of children. However, due to the fact that the microarrays used in our study (Infinium CoreExome-24 v1-4, Illumina) were not fully compatible with those used by Kühnisch et al. (Affymetrix Human SNP Array 5.0 or 6.0, Affymetrix, Santa Clara, CA, USA), we were able to replicate assessments only for three out of five MIH-suggestive loci identified in German children from the GINI-plus and LISA-plus cohorts, i.e., for loci at chromosomes 9 (rs2889956), 20 (rs4811117), and 22 (rs13058467) [12]. However, there were no significant associations between rs2889956, rs4811117, or rs13058467 and susceptibility to molar incisor hypomineralization in Polish children. A lack of an association between each SNP and MIH was found in analyses performed for frequency distributions of major homozygotes, heterozygotes, and minor homozygotes (except for the rs4811117 polymorphism, for which the *p*-value reached marginal significance at *p* = 0.017, which is on the borderline if the Bonferroni-corrected significance level of *p* = 0.05/3 = 0.017 is used for the global chi-squared test with the further tests treated as post-hoc tests).

As a matter of interest, we decided to determine whether the significant effects found by Kühnisch et al. [12] would disappear if our data were added to the data presented by Kühnisch et al. obtained from a group of German children, in other words by creating a combined dataset, ignoring possible systematic differences between the two studies. Our analysis performed for rs4811117 and rs13058467 in this combined group revealed that the significant associations for both SNPs with predisposition to MIH remained in all modes of inheritance, with the lowest *p*-value (4 × 10^−7^) for rs13058467 near the *SCUBE1 locus* (Table S1 in Supplementary Materials). 

Similar to Kühnisch et al. [12], in our analyses we did not apply adjustments for sex and age because there were no statistically significant differences in these variables between the MIH and non-MIH groups. The results of a recent meta-analysis by Mazur et al. revealed a significant correlation between MIH and caries in permanent dentition in children [17]. However, a few researchers have not confirmed this [18,19]. Additionally, in our study, there were no significant differences in caries prevalence between the MIH and non-MIH groups; therefore, no adjustment for caries was performed.

Despite the fact that our study group consisted of 110 children more than the cohort of German children analyzed in the GWAS by Kühnisch et al. [12], we did not replicate the significant associations of rs2889956, rs4811117, or rs13058467 with MIH in permanent dentition in Polish children. We suggest lower MIH prevalence in Polish children and differences in SNP genotype frequencies between the studies as potential causes of the lack of confirmation of these results.

In a GWAS published in 2014, Kühnisch et al. found that 13.2% of children (average age 10.2 years, standard deviation 0.2 years) from their GINI-plus and LISA-plus cohorts recruited in Munich were affected by molar incisor hypomineralization [12]. However, in older children, MIH was detected in 17.2% subjects from both cohorts examined at the age of 15 years [20]. In addition, epidemiological data for the 2014–2015 school year reported by Amend et al. showed that the MIH prevalence in children (6 to 12 years old) was 9.4% in a rural area of Central Hesse as compared to 17.4% in Frankfurt on the Main, indicating regional variations probably associated with the geographical area of permanent residence (rural versus urban) [21]. 

It is worth noting that 10.2% of our group (children aged 126–168 months and recruited between 2019 and 2022) had MIH. In contrast, in 2019, Glodkowska and Emerich reported an MIH frequency of 6.43% in 6- to 12-year-old children (*n* = 1437) living in northern Poland [22], and Ilczuk-Rypuła et al. in 2022 reported an MIH prevalence in Silesian children (9.0 ± 1.9 years old) of 6.2% [23].

The frequencies of the potential risk alleles of rs4811117 or rs13058467 polymorphisms in our group, as compared with German cohort, were 4.2% or 2.2% lower, respectively. In contrast, the prevalence of the rs2889956 minor A allele in Polish children was 5.4% higher as compared with its frequency in German children. These differences provide some further evidence that despite being adjacent geographically, there can be significant genetic population differentiation between the two countries [24]. Note that the Sorbs of Slavic descent in the Lusatia region of eastern Germany have previously shown the greatest genetic similarity to other West Slavs, such as Poles or Czechs, and possible analyses using this group might provide a way of disentangling genetic from environmental differences between German and Slavic populations [25].

Among five loci, the most significantly associated SNP with MIH was found by Kühnisch et al. [12] to be rs13058467, located near the *SCUBE1* gene that encodes the protein Signal peptide, CUB and EGF-like domain-containing protein 1. This gene has been shown to be involved in tooth development [26]. However, the suggestion that rs13058467 affects *SCUBE1*-related functional significance seems to be inconsistent with data from Ensembl (www.ensembl.org, accessed on 20 January 2024) indicating that this SNP results in a change from asparagine (Asn) to serine (Ser) at amino acid 95 (p.Asn95Ser) in the *TTLL12* gene encoding tubulin-tyrosine ligase-like protein 12. In addition, the p.Asn95Ser amino acid substitution seems to be without any pathogenic impact on the structure and function of the protein encoded by the *TLL12* gene. This is shown so far by all in silico tools linked to Ensembl, and the results indicate that the mutation is tolerated (SIFT and MetaLR), benign (PolyPhen), likely benign (CADD and REVEL), or neutral (Mutation Assessor). In turn, both rs2889956 and rs4811117 are non-coding variants. This phenomenon is very common and concerns as many as 90% of genetic polymorphisms associated to date with particular phenotypes or diseases in genome-wide association studies [27]. It is also worth noting that for both SNPs, there are no candidate genes associated with tooth phenotypes in their physical proximity (±100 kb windows) at the chromosome 9 or chromosome 20, respectively.

We are fully aware that, as in the GWAS from Kühnisch et al. [12], a major limitation of our study that limits reliable conclusions is its relatively low statistical power. Our statistical under-powering results mainly from a relatively small sample size even though our study is 110 subjects greater than the population analyzed in the GWAS by Kühnisch et al. [12]. Their study was based on 88 MIH cases in a cohort consisting of 668 children (with MIH prevalence 13.2%) and could only detect very large genetic effect sizes ranging from an odds ratio (OR) of 4.1 for a minor allele frequency of 10% to an OR of 2.9 for a minor allele frequency of 30% (using a significance level of 5 × 10^−8^ with a power of >80%; in fact, the frequency of the C allele of the most significant, rs13058467, polymorphism was 10.4%) [12]. The authors also emphasized that a similar study able to detect effect sizes at an OR = 1.2 using the same significance level with the same power would require about 7000 cases for a minor allele frequency of 10% assuming the same MIH population prevalence. 

The Open Epi (www.openepi.com) software (OpenEpi: Open Source Epidemiologic Statistics for Public Health, updated 2013/04/06, accessed on 18 December 2023) for epidemiologic statistics was used to compute the minimum sample size for 80% statistical power and a 5% type I error rate (α). We assumed a ratio of non-MIH to MIH subjects equal to 10.44 (710/68) and a frequency of subjects with at least one C allele of rs13058467 equal to 15.5% in the non-MIH group and 22.14% in the MIH group (using a dominant mode of inheritance). Under these assumptions, the estimated confidence interval for the minimum sample size needed was 3015 to 3148 children (with 264 to 299 MIH cases). An additional cause for statistical under-powering in our study involves the differences in prevalence of the analyzed polymorphisms between our cohort of Polish children and the German children included in the GWAS. The frequencies of suggested MIH risk (minor) alleles of rs4811117 or rs13058467 in our study were significantly lower as compared with the GWAS data (T allele of rs4811117: 8.2% versus 10.4%, *p* < 0.05; or C allele of rs13058467: 8.2% versus 12.4%, *p* < 0.002). In the present study, we assessed children born in Szczecin, descendants of residents of Polish West Pomerania who migrated to this region (after World War II) from other Polish regions [24,28]. Contemporary residents of this region can therefore be considered representative for the entire Polish population [29,30]. The SNP frequencies determined in this study can be treated as values likely corresponding to the prevalence in the Polish population in general.

Taking into consideration the above facts and our own experience, we strongly recommend that further research on the genetic predisposition to MIH should be based on genome-wide association studies in large groups of subjects. Significant associations found in only one ethnic group need to be replicated in other groups. Subsequently, genetic variants identified in GWAS must assessed for actual functional significance [27].

In conclusion, our replication study in Polish children did not confirm highly significant associations of molar incisor hypomineralization with rs2889956, rs4811117, or rs13058467, which were previously suggested to be possibly linked to MIH using a significance threshold *p*-value of <1 × 10^−6^ in a GWAS that assessed German children. Taking into account possible unknown systematic differences in the way that the studies were conducted, there is still a slight possibility that rs4811117 has a minor association that should be investigated further.

## Figures and Tables

**Table 1:** Basic characteristics of studied children in regard to molar incisor hypomineralization (MIH).

Variable	Whole Group (*n* = 778)	MIH Group (*n* = 68)	Non-MIH Group (*n* = 710)	*p*
Median Age (months), [minimum–maximum]	152 (126–168)	153 (132–168)	152 (126–168)	0.481 ^a^
Sex: female/male, (*n* [%])	362/416 (46.5/53.5)	34/34 (50.0/50.0)	328/382 (46.2/53.8)	0.549 ^b^
DMFT: 0/≥1, (*n* [%])	308/470 (39.6/60.4)	29/39 (42.6/57.4)	279/431 (39.3/60.7)	0.590 ^b^

DMFT: Decayed Missing Filled Teeth Index; ^a^ Mann-Whitney U test; ^b^ Chi-squared test.

**Table 2:** Association analyses of rs2889956, rs4811117, and rs13058467 genetic polymorphisms with molar incisor hypomineralization in Polish children.

Polymorphism	Allele ^b^	Distribution of Alleles, *n* [%]		*p*	Distribution of Genotypes, *n* [%]						*p*	*p*_d_	*p*_r_	*p*_a_
(Location) ^a^	(1/2)	MIH Group	Non-MIH Group		MIH Group			Non-MIH Group						
		1/2	1/2		1;1	1;2	2;2	1;1	1;2	2;2				
rs2889956 (9:2889956)	G/A	95/41 (69.8/30.2)	989/431 (69.6/30.4)	0.962	32 (47.1)	31 (45.6)	5 (7.3)	348 (49.0)	293 (41.3)	69 (9.7)	0.708	0.759	0.527	0.960
rs4811117 (20:5105498)	G/T	129/7 (94.8/5.2)	1299/121 (91.5/8.5)	0.229	62 (91.2)	5 (7.3)	1 (1.5)	590 (83.1)	119 (16.8)	1 (0.1)	0.017	0.091	0.097	0.165
rs13058467 (22:43183043)	T/C	120/16 (88.2/11.8)	1308/112 (92.1/7.9)	0.159	53 (77.9)	14 (20.6)	1 (1.5)	600 (84.5)	108 (15.2)	2 (0.3)	0.155	0.162	0.177	0.112

^a^ Single nucleotide polymorphism location was indexed to NCBI build 38 (GRCh38.p13). ^b^ Alleles 1 and 2 were defined as the major and minor (rarer) alleles, respectively. *p*—significance values for chi^2^ 2 × 2 table (alleles) or for chi^2^ 2 × 3 table (genotypes). *p*_d_, *p*_r_ or *p*_a_—significance values for logistic regression in dominant, recessive, or additive modes of inheritance for the minor allele (allele 2), respectively. No adjustment for age, sex, and caries was necessary because those covariates were found not to be associated with MIH.

## Data Availability

The data are available from the corresponding author upon reasonable request during the study.

## Supplementary Materials

The following supporting information can be downloaded at: https://www.mdpi.com/article/10.3390/jcm13030857/s1, Table S1. Association analyses of rs4811117 and rs13058467 genetic polymorphisms with molar incisor hypomineralization in combined groups consisting of German and Polish children.
