# Supplementary material for: Association of Three Genetic Loci with Molar Incisor Hypomineralization in Polish Children
Source: J Clin Med. 2024 Feb 1;13(3):857. doi: 10.3390/jcm13030857 (PMC10856562; doi:10.3390/jcm13030857)
Supplement: Supplementary file 1 [file jcm-13-00857-s001.zip › jcm-2826689-supplementary.pdf]

Table S1. Association analyses of rs4811117 and rs13058467 genetic polymorphisms with molar-incisor hypomineralization in combined group consisted of German and Polish children.

Polymorphism (location)[a]	**Allele[b]** (1/2)	**Distribution of alleles, n [%]** MIH group 1/2	Non-MIH group 1/2	p	**Distribution of genotypes, n [%]** MIH group (n=156) 1;1	1;2	2;2	Non-MIH group (n=1290) 1;1	1;2	2;2	**p**	**p_d**	**p_r**	p_a
rs4811117 (20:5105498)	G/T	265/47 (85.9/15.1)	2333/247 (90.4/9.6)	0.003	112 (71.8)	41 (26.3)	3 (1.9)	1049 (81.3)	235 (18.2)	6 (0.5)	0.004	0.006	0.045	0.003
rs13058467 (22:43183043)	T/C	260/52 (83.3/16.7)	2365/215 (91.7/8.3)	<0.00001	110 (70.5)	40 (25.7)	6 (3.8)	1082 (83.9)	201 (15.6)	7 (0.5)	<0.00001	0.00005	0.0005	0.0000004

[a] Single nucleotide polymorphism location was indexed to NCBI build 38 (GRCh38.p13).

[b] Alleles 1 and 2 were defined as the major and minor (rarer) alleles, respectively.

p – significance values for chi^2 2x2 table (alleles) or for chi^2 2x3 table (genotypes).

p_d, p_r or p_a – significance values for logistic regression in dominant, recessive or additive mode of inheritance for minor allele (allele 2), respectively. No adjustment for age and sex was necessary because those covariates were found not to be associated with MIH.
